# Corticofugal modulation of peripheral auditory responses

**DOI:** 10.3389/fnsys.2015.00134

**Published:** 2015-09-30

**Authors:** Gonzalo Terreros, Paul H. Delano

**Affiliations:** ^1^Programa de Fisiología y Biofísica, ICBM, Facultad de Medicina, Universidad de ChileSantiago, Chile; ^2^Departamento de Otorrinolaringología, Hospital Clínico de la Universidad de ChileSantiago, Chile

**Keywords:** auditory efferent, olivocochlear, top-down, neural network, descending projections, corticofugal

## Abstract

The auditory efferent system originates in the auditory cortex and projects to the medial geniculate body (MGB), inferior colliculus (IC), cochlear nucleus (CN) and superior olivary complex (SOC) reaching the cochlea through olivocochlear (OC) fibers. This unique neuronal network is organized in several afferent-efferent feedback loops including: the (i) colliculo-thalamic-cortico-collicular; (ii) cortico-(collicular)-OC; and (iii) cortico-(collicular)-CN pathways. Recent experiments demonstrate that blocking ongoing auditory-cortex activity with pharmacological and physical methods modulates the amplitude of cochlear potentials. In addition, auditory-cortex microstimulation independently modulates cochlear sensitivity and the strength of the OC reflex. In this mini-review, anatomical and physiological evidence supporting the presence of a functional efferent network from the auditory cortex to the cochlear receptor is presented. Special emphasis is given to the corticofugal effects on initial auditory processing, that is, on CN, auditory nerve and cochlear responses. A working model of three parallel pathways from the auditory cortex to the cochlea and auditory nerve is proposed.

## Introduction

Since the first description of the crossed and uncrossed olivocochlear (OC) bundles by Rasmussen (1946, 1960), these brainstem pathways have been considered as the auditory efferent system itself, and the terms “*olivocochlear*” and “*auditory efferent*” have been frequently used as synonyms. However, several lines of neuroanatomical evidence demonstrate the presence of an efferent network originated in the auditory cortex that reaches the cochlear receptor through OC neurons (Feliciano et al., 1995; Mulders and Robertson, 2000). This network comprises descending projections from the auditory cortex to the medial geniculate body (MGB), inferior colliculus (IC), cochlear nucleus (CN) and superior olivary complex (SOC) that form multiple feedback loops, including the: (i) colliculo-thalamic-cortico-collicular; (ii) cortico-(collicular)-OC; and (iii) cortico-(collicular)-CN pathways (Saldaña et al., 1996; Robles and Delano, 2008; Xiong et al., 2009; Malmierca and Ryugo, 2011; Schofield, 2011).

The functionality of the corticofugal pathways to OC neurons has been proven by recent evidence demonstrating that auditory cortex activity can modulate afferent responses even at the level of sensory transduction (Xiao and Suga, 2002; León et al., 2012). Several functions have been attributed to these corticofugal effects on cochlear responses, including selective attention (Oatman, 1971), modulation of afferent inputs during wake/sleep cycle (Velluti et al., 1989) and antimasking of acoustic signals in noise background (Nieder and Nieder, 1970). It is important to highlight that any efferent modulation of the most peripheral structures of the auditory pathway—the auditory nerve and cochlear hair cells—must be mediated by the OC system.

In this mini-review, anatomical and physiological evidence supporting the presence of a functional efferent network from the auditory cortex to the cochlear receptor are presented. Special emphasis is given to the corticofugal effects on CN, auditory nerve and cochlear responses produced by auditory cortex manipulations. A working model of three parallel pathways from the auditory cortex to the cochlea and auditory nerve is proposed.

## The Olivocochlear System: the Final and Mandatory Pathway from the Central Nervous System to the Cochlear Receptor

The OC system comprises medial (MOC) and lateral (LOC) olivocochlear neurons located in the SOC (Warr and Guinan, 1979). MOC neurons have thick and myelinated axons that are mainly directed towards outer hair cells (OHC) of the contralateral cochlea, while LOC neurons possess thin and unmyelinated fibers that make synapses with ipsilateral auditory nerve dendrites just beneath cochlear inner hair cells (Guinan, 1996). Similarly to alpha-motor neurons, MOC neurons release acetylcholine as their main neurotransmitter, and activate nicotinic receptors comprised by five α9/α10 subunits located in OHCs (Elgoyhen et al., 1994, 2001). On the other hand, several neurotransmitters and neuromodulators are known to be released by LOC neurons, including acetylcholine, GABA, dopamine, dynorphines, encephalin, and CGRP (Eybalin, 1993). Importantly, for the central nervous system, MOC neurons are the final and mandatory pathway to regulate the mechanical vibrations of the basilar membrane, acting as individual motor units along the cochlear partition (LePage, 1989; Murugasu and Russell, 1996; Cooper and Guinan, 2003). Therefore, the OC system is fundamental in the functioning of the efferent network, as all cortical or subcortical modulations of cochlear and auditory nerve responses must be transmitted through MOC or LOC synapses.

The OC function can be evaluated through a brainstem reflex that is activated by ipsilateral or contralateral auditory stimulation (Buño, 1978; Liberman, 1989). The neural circuit of this reflex is constituted by auditory nerve fibers, CN neurons, and crossed or uncrossed MOC fibers (de Venecia et al., 2005). The ipsilateral MOC reflex pathway comprises a double crossing, including the afferent pathway from the CN and the crossed MOC fibers, while the contralateral MOC reflex comprises only one crossing in the ascending pathway and the uncrossed MOC fibers. There is also anatomical evidence showing differences in the cochlear innervation patterns of crossed and uncrossed MOC fibers (Brown, 2014), which is in agreement with physiological data obtained in humans, that suggest different functions for the crossed and uncrossed MOC reflex (Lilaonitkul and Guinan, 2009). In addition, indirect LOC stimulation through IC descending pathways modulates the amplitude of auditory nerve responses (Groff and Liberman, 2003). Therefore, the OC system can modulate OHC and auditory nerve responses through three different pathways: (i) the crossed; (ii) uncrossed MOC fibers; and (iii) LOC neurons.

## Descending Projections from the Auditory Cortex to the Medial Geniculate Body

Among the auditory subcortical nuclei, the MGB receives the largest number of cortical descending projections from pyramidal neurons located in layers V and VI of the auditory cortex, forming tonotopic feedback loops between the primary auditory cortex and ventral MGB (Bartlett et al., 2000; Winer et al., 2001; Winer, 2006; Winer and Lee, 2007). Physiological studies demonstrate that the auditory cortex modulates MGB responses (Ryugo and Weinberger, 1976; Villa et al., 1991; Zhang and Suga, 2000; Antunes and Malmierca, 2011), and that this modulation is different for ventral and medial MGB neurons (Tang et al., 2012). The electrical microstimulation of the auditory cortex produced sharply tuned effects in the ventral MGB, while suppressive and broad-band effects were obtained in the medial MGB. However, whether the corticofugal modulation of thalamic neurons affects OC activity is unknown. Importantly, as there is no anatomical evidence of direct descending connections from MGB to OC neurons, any possible thalamic modulation of OC activity should be produced indirectly through the colliculo-thalamic-cortico-collicular loop.

## Descending Projections from the Auditory Cortex to the Inferior Colliculus, Superior Olivary Complex and Cochlear Nucleus

The IC is a key structure of the ascending and descending auditory pathways (Huffman and Henson, 1990). Direct descending projections from the auditory cortices to the IC are mainly originated in layer V of the primary fields, and in a lesser extent from layer VI (Faye-Lund, 1985; Doucet et al., 2003). Most of the cortico-collicular fibers are glutamatergic (Feliciano and Potashner, 1995) and are directed to the ipsilateral IC, but there are also fibers directed to the contralateral IC (Bajo et al., 2007; Nakamoto et al., 2013a,b). Although the majority of cortico-collicular descending projections are directed to the IC cortices, a small subset reaches the central nucleus of the IC, which is the main ascending and tonotopic structure of this nucleus (Saldaña et al., 1996; Bajo and Moore, 2005). In agreement with these neuroanatomical findings, physiological evidence demonstrates a tonotopic modulation of the central nucleus of the IC by auditory cortex microstimulation (Yan and Suga, 1998; Yan et al., 2005). In addition, IC responses to sound intensity (Yan and Ehret, 2002), duration (Ma and Suga, 2001) and location (Zhou and Jen, 2005) are also modulated by the auditory cortex. More comprehensive reviews about the corticofugal effects on IC responses can be found elsewhere (Anderson and Malmierca, 2013; Bajo and King, 2013; Malmierca et al., 2015).

**Figure 1:**
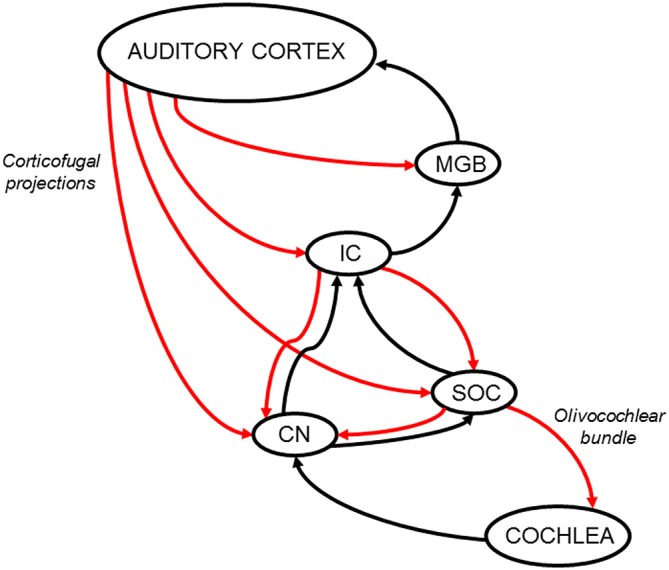
**Schematic diagram of the auditory efferent network.** Ascending and descending pathways are depicted in black and red arrows respectively. A simplified model of the auditory efferent system is presented. Corticofugal projections from the auditory cortex to the inferior colliculus (IC) and medial geniculate body (MGB) and afferent connections from the IC and MGB to the auditory cortex form a “top loop” within this network (colliculo-thalamic-cortico-collicular loop, Xiong et al., 2009). Bottom loops are constituted by auditory-cortex descending projections to the CN (cortico-(collicular)-cochlear nucleus loop) and SOC (cortico-(collicular)-olivocochlear loop), which are connected to the cochlear receptor by the OC bundle. Note, the relevant position of the IC in the interaction between top and bottom loops. In addition, it is important to highlight that cortical modulations of cochlear responses, can modify IC responses through ascending connections to the CN and IC, meaning that the interaction between the top and bottom loops is bidirectional. CN: cochlear nucleus; IC: inferior colliculus; MGB: medial geniculate body; SOC: superior olivary complex.

The SOC and the CN are also direct targets of cortical descending projections, mainly from primary auditory cortex, but also from ventral and rostral secondary fields (Weedman and Ryugo, 1996a, b; Doucet et al., 2002). Moreover, Mulders and Robertson showed evidence of the presence of synaptic connections between cortical descending axons and MOC neurons (Mulders and Robertson, 2000). There is also evidence of indirect connections between the auditory cortex and SOC through IC synapses (Thompson and Thompson, 1993; Vetter et al., 1993). Despite these neuroanatomical findings, there is still no physiological evidence of how the auditory cortex modulates the activity of SOC neurons. Regarding connections from the auditory cortex to the CN, Schofield and colleagues have demonstrated that the dorsal and ventral CN receive direct projections from the auditory cortex, as well as indirect projections, passing through the IC or the SOC (Schofield and Cant, 1999; Schofield and Coomes, 2005; Schofield et al., 2006). In summary, descending projections from the auditory cortex to the IC, SOC and CN create multiple feedback loops that can modulate cochlear responses through OC neurons (Figure 1).

## Corticofugal Effects on Cochlear Nucleus

The first evidence of a feedback control of CN responses by the central nervous system was found several decades ago in physiological experiments performed in awake and behaving cats (Hernández-Peón et al., 1956; Dewson et al., 1966). Hernández-Peón et al. (1956) found a reduction in the evoked potentials recorded from the CN in cats while receiving stimuli of other sensory modalities. Later, Dewson et al. (1966) showed that auditory cortex ablations modified the evoked responses of the CN. These pioneer studies suggested the presence of corticofugal pathways from the auditory cortex to the CN that were discovered several decades later (Weedman and Ryugo, 1996a, b; Schofield and Coomes, 2005, 2006).

The functionality of these pathways has been recently confirmed by studying the effects of electrical microstimulation of the auditory cortex in the contralateral CN of mice (Luo et al., 2008). These authors found that focal stimulation of a specific area of the auditory cortex increased the magnitudes and shortened the latencies of the responses of ventral CN neurons that had similar characteristic frequencies to the stimulated cortical site, while opposite effects were observed for CN neurons with other characteristic frequencies (Luo et al., 2008). Moreover, they found similar results in the ipsilateral ventral CN (Liu et al., 2010) and in the dorsal CN (Kong et al., 2014). These physiological studies are illustrative of a general characteristic of the efferent system: the frequency selectivity of corticofugal projections, which has also been obtained activating the descending pathways to the MGB, IC and cochlea (Suga and Ma, 2003).

## Corticofugal Effects on Auditory-Nerve and Cochlear Responses

Only a few physiological studies have assessed the corticofugal effects of auditory cortex manipulations on the most peripheral auditory structures, including, auditory nerve responses (León et al., 2012; Dragicevic et al., 2015), cochlear electrical responses (Xiao and Suga, 2002; León et al., 2012; Dragicevic et al., 2015) and otoacoustic emissions (OAE; Khalfa et al., 2001; Perrot et al., 2006). In a seminal work, Xiao and Suga (2002) demonstrated that the auditory cortex activity modulates the amplitude and frequency tuning of cochlear microphonics (CM) responses near the echolocalizing frequency of the mustached bat (61 kHz). In addition, corticofugal effects on cochlear responses have been found in human patients with epilepsy refractory to pharmacological treatment. In these patients, cortical resection of the temporal superior gyrus produced a bilateral reduction of the MOC reflex that was more pronounced in the ear contralateral to the resected auditory cortex (Khalfa et al., 2001). Moreover, electrical microstimulation of the auditory cortex by means of a chronic intra-cerebral multielectrode array produced a significant reduction of OAE, while there was no change under stimulation of non-auditory cortical areas (Perrot et al., 2006).

A recent work by León et al. (2012) extended the findings of the corticofugal effects observed in bats and humans to the chinchilla. In this work the spontaneous activity of the auditory cortex was inactivated by two methods: cortical cooling with cryoloops and lidocaine microinjections. The combined experimental approaches and the adequate control of the cochlear temperature ruled out the possibility of a direct cooling of the cochlea by the cortical cryoloops, as it has been suggested in guinea pigs by Coomber et al. (2011). Both types of cortical manipulations produced changes in the amplitudes of auditory-nerve compound action potentials (CAP) and CM responses (León et al., 2012). Although these effects were diverse, the most common pattern was a concomitant reduction in the amplitudes of CAP and CM responses. The electrical stimulation of the crossed MOC fibers at the floor of the fourth ventricle produces a decrease in CAP amplitudes and a simultaneous increase of CM responses (Gifford and Guinan, 1987). Therefore, the parallel changes of CAP and CM amplitudes observed in chinchillas after auditory cortex inactivation suggest a concomitant modulation of MOC and LOC neurons, as the latter system can only affect CAP but not CM responses (Groff and Liberman, 2003). León et al. (2012) proposed that the ongoing activity of the auditory cortex regulates cochlear sensitivity through parallel pathways to the cochlear receptor. However, whether these corticofugal effects were affecting the functioning of the OC reflex circuit remained unknown.

**Figure 2:**
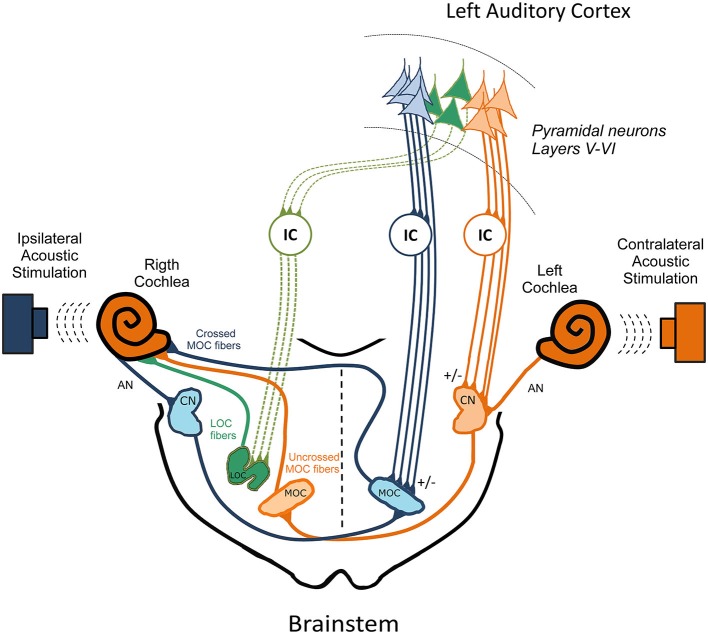
**The three pathways model for the cortico-collicular-olivocochlear and cochlear nucleus circuits.** In order to simplify this model, the colliculo-thalamic-cortico-collicular loop has been omitted. In addition, only efferent pathways from the left auditory cortex to the right cochlea are presented. Three OC pathways are directed to the right cochlear receptor and auditory nerve, which are depicted in color green, orange and blue corresponding to the: (i) right LOC fibers; (ii) right uncrossed MOC; and (iii) left crossed MOC neurons respectively. Ipsilateral acoustic stimulation of the right cochlea activates right AN, right CN neurons that send projections to the contralateral MOC. In turn, left crossed MOC neurons modulate right cochlear responses (blue brainstem pathways), constituting the ipsilateral OC reflex. On the other hand, contralateral acoustic stimulation of the left cochlea activates, left AN, left CN neurons that send projections to the right uncrossed MOC fibers, which modulate right cochlear responses (orange brainstem pathways), constituting the contralateral OC reflex that connects both ears. This model proposes that the descending pathways from the left auditory cortex directed to the left IC and to the left CN (orange corticofugal pathways) modulate the contralateral OC reflex, by regulating the activity of the left CN and right uncrossed MOC neurons. On the other hand, descending pathways directed to the left IC and left MOC (blue corticofugal pathways) regulate crossed MOC activity, which is involved in the ipsilateral OC reflex. Finally, corticofugal pathways to the contralateral IC (green corticofugal pathways) could regulate right LOC neurons, modulating the activity of right AN fibers. The +/− signs represent possible excitatory and inhibitory pathways. Modified from Dragicevic et al. (2015) with permission. AN: auditory nerve; CN: cochlear nucleus; LOC: lateral olivocochlear; MOC: medial olivocochlear; IC: inferior colliculus.

In a recent study, Dragicevic et al. (2015) used auditory cortex microstimulation in chinchillas to demonstrate that in addition to the corticofugal modulation of cochlear sensitivity on CAP and CM responses, the auditory cortex also modulates the strength of the contralateral OC reflex on CAP but not on CM responses. In agreement with neuroanatomical data, the largest corticofugal effects were obtained in auditory cortices with short latency responses (<15 ms), which correspond to primary auditory fields. Moreover, these two types of corticofugal modulations: (i) on cochlear sensitivity; and (ii) on the OC reflex strength were not correlated, suggesting the presence of at least two functionally different descending pathways to the crossed and uncrossed MOC neurons, and possibly a third pathway to LOC neurons (Figure 2). Some functional consequences of the corticofugal effects on the strength of the OC reflex could be the finding of stronger reflexes in awake than in anesthetized animals (Guitton et al., 2004; Chambers et al., 2012; Aedo et al., 2015), and the diminishing of tinnitus perception during stimulation of the auditory cortex in human patients (Fenoy et al., 2006; Fregni et al., 2006).

The model of three parallel descending pathways from the auditory cortex, is also supported by the presence of different types of projecting neurons, including regular and burst spiking pyramidal neurons from layer V (Hefti and Smith, 2000), and neurons from layer VI (Winer et al., 2001). Moreover, the differential corticofugal effects obtained with different microstimulation rates: 5 Hz to modulate IC and MGB responses (Suga and Ma, 2003) and 32–33 Hz to modulate SOC activity (Xiao and Suga, 2002; Dragicevic et al., 2015), suggest different activation thresholds for cortical neurons projecting to these subcortical nuclei.

## Functional Role of the Auditory Efferent System

Different functions can be assigned to the different loops formed in the auditory efferent pathways. Functions mainly depending on the OC brainstem circuit are protection to acoustic trauma (Maison and Liberman, 2000) and balance of interaural cochlear sensitivity (Darrow et al., 2006), while neural plasticity during learning of behaviorally relevant auditory tasks has been attributed to the colliculo-thalamic-cortico-collicular loop (Xiong et al., 2009; Bajo et al., 2010). A top-down frequency filter needed in different behavioral situations can be proposed as the general function for the cortico-olivocochlear circuit, including selective attention to auditory or visual stimuli (Delano et al., 2007; Smith et al., 2012), regulation of afferent responses during wake/sleep cycle (Velluti et al., 1989; Froehlich et al., 1993), and antimasking of auditory stimuli in a noisy environment (Kawase and Liberman, 1993). As there is increasing evidence of the modulation of cochlear responses during selective attention, this putative function of the corticofugal system is discussed next.

## Selective Attention to Visual or Auditory Stimuli

Since the early experiments performed in cats by Hernández-Peón et al. (1956), the auditory efferent system has been proposed to function as a top-down filter of peripheral auditory responses during attention. To address this proposal, two types of attentional paradigms have been used: (i) attention to visual stimuli using irrelevant auditory distractors (Oatman, 1971; Delano et al., 2007), in which all peripheral auditory responses at all frequencies should be suppressed through the efferent system; and (ii) attention to auditory targets of specific frequency (Smith et al., 2012; Srinivasan et al., 2012), in which the peripheral auditory responses near the target frequency would be enhanced while other frequencies would be suppressed by the efferent system.

CAP reductions in response to click and tone auditory distractors during selective attention to visual stimuli have been obtained in cats and chinchillas (Oatman, 1971; Delano et al., 2007). In the latter work, CM increases concomitant to CAP reductions were obtained during visual attention, suggesting that these attentional effects were indeed produced by activation of MOC neurons, as the electrical stimulation of MOC fibers produces CAP reductions with simultaneous CM increases (Elgueda et al., 2011).

Contradictory results have been obtained in visual attention tasks with auditory distractors in humans. For instance, Puel et al. (1988) showed that click-evoked OAE were reduced in 13 out of 16 evaluated subjects during visual attention (1.25 dB in average). Similarly, Wittekindt et al. (2014) showed that during periods of visual attention to Gabor patches, there was a reduction in the amplitude of distortion product otoacoustic emissions (DPOAE). On the other hand, Smith and colleagues (Smith et al., 2012; Srinivasan et al., 2012), found a DPOAE increase during selective attention to visual stimuli, but a DPOAE reduction during auditory attention to the DPOAE primary tones (f_1_ and f_2_). These opposite results could be explained by the differential generating mechanisms of click-evoked OAE with that of DPOAEs (Shera and Guinan, 1999). Importantly, in the work of Smith et al. (2012), subjects attended to the primary tones (f_1_ and f_2_) that generate the DPOAEs, but measurements were obtained from a distant location in the cochlear partition at the 2f_1_–f_2_ frequency. Future experiments should clarify whether the corticofugal effects are different if the subject attention is directed to the primary tones (f_1_ and f_2_) or to the DPOAE frequency (2f_1_–f_2_).

Both ears are connected through the uncrossed MOC pathway that is activated by contralateral sounds (de Venecia et al., 2005). Notably, differential effects of interaural attention through uncrossed MOC fibers have been found in human studies (de Boer and Thornton, 2007; Srinivasan et al., 2014). Srinivasan et al. (2014) found that alternating auditory attention between the two ears modifies the strength of corticofugal effects on DPOAEs responses, suggesting that attention can independently modulate crossed and uncrossed MOC neurons. These findings are in agreement with the results obtained by Dragicevic et al. (2015) and support the model proposed in this article (Figure 2).

## Concluding Remarks

In summary, here we reviewed the growing anatomical and physiological evidence supporting the presence of an efferent network from auditory cortex to OC neurons. Cortical descending effects on CN, auditory nerve and cochlear responses are proposed to be produced by three parallel pathways from auditory cortex to the crossed and uncrossed MOC neurons and to LOC neurons. These connections are probably activated during selective attention, learning induced plasticity and other cognitive functions.

## Conflict of Interest Statement

The authors declare that the research was conducted in the absence of any commercial or financial relationships that could be construed as a potential conflict of interest.
